# Temporal Variation and Industry-Specific Differences of the Use of Volatile Organic Compounds from 2018 to 2023 and Their Health Risks in a Typical Industrially Concentrated Area in South China

**DOI:** 10.3390/toxics12090634

**Published:** 2024-08-29

**Authors:** Yijia Guo, Lihua Zhu, Liyin Zhang, Xinxin Tang, Xinjie Li, Yiming Ge, Feng Li, Jilong Yang, Shaoyou Lu, Jinru Chen, Xiaotao Zhou

**Affiliations:** 1 Public Health Service Center, Bao’an District, Shenzhen 518126, China; beckyguo1995@126.com (Y.G.); zhuivy810@126.com (L.Z.); 13798509754@126.com (L.Z.); lf13424232209@163.com (F.L.); yangjlong6@mail2.sysu.edu.cn (J.Y.); 2 School of Public Health (Shenzhen), Shenzhen Campus of Sun Yat-sen University, Shenzhen 518107, China; tangxx26@mail2.sysu.edu.cn (X.T.); lixj328@mail2.sysu.edu.cn (X.L.); geym5@mail2.sysu.edu.cn (Y.G.); lushy23@mail.sysu.edu.cn (S.L.); 3 School of Public Health, Sun Yat-Sen University, Guangzhou 510080, China

**Keywords:** organic solvents, industries, occupational hazards, Shenzhen

## Abstract

The risk of occupational exposure to organic solvents varies across industries due to factors such as processing materials, ventilation conditions, and exposure duration. Given the dynamic nature of organic solvent use and occupational exposures, continuous monitoring and analysis are essential for identifying high-risk hazards and developing targeted prevention strategies. Therefore, this study aims to analyze the use of organic solvents and volatile organic compounds (VOCs) in different industries in Bao’an District, Shenzhen, China, from 2018 to 2023, to understand their temporal variation and industry-specific differences and to identify high-risk occupational hazards. This study includes 1335 organic solvent samples, used by 414 different industry enterprises, and 1554 air samples. The result shows that the usage of organic solvents in various industries decreased with the outbreak of the pandemic and, conversely, increased as the situation improved. The most frequently detected volatile components in organic solvents were alkanes, followed by aromatic hydrocarbons. The ratios of the detection frequency of VOCs to the total number of detected categories increased year by year after 2020, indicating a tendency towards reduction and concentration of the types of organic solvents used in industrial production. Among the 8 high-risk VOCs, toluene (22.5%), n-hexane (22.0%), xylene (16.1%), and ethylbenzene (15.3%) have relatively high detection rates, suggesting that they need to be focused on in occupational health. Through air samples, the results show that trichloroethylene and xylene pose a high risk to human health (HQ > 1). We recommend that industry should strengthen monitoring of these two VOCs.

## 1. Introduction

Organic solvents, integral to industrial processes [1], find widespread application across various sectors, including paints, coatings, adhesives, and cleaners [2,3,4]. They serve not only as solvents for dissolving raw materials and facilitating reactant cross-linking but also contribute to enhancing the performance, stability, and durability of the resulting products. Nonetheless, their extensive utilization carries inherent environmental implications that warrant attention [5]. During industrial operations, the volatilization of organic solvents releases harmful gases, contributing to atmospheric pollution. These emissions give rise to pollutants, such as ozone and nitrogen dioxide, detrimental to both human health [6,7,8] and vegetation [9]. Furthermore, organic solvents can infiltrate water bodies and soil through various pathways, posing risks to water resources and soil integrity, ultimately impacting biodiversity and ecological equilibrium [10]. In summary, organic solvents play an indispensable role in industrial production, but their potential impact on the environment should not be ignored.

Volatile organic compounds (VOCs) constitute the primary components of the organic solvents widely utilized in industrial processes [11]. However, their volatile nature renders VOCs a significant source of environmental contamination and occupational health hazards [12,13,14]. Through extensive testing of organic solvent samples, it has been observed that VOCs exhibit a diverse range of types and concentrations, underscoring their notable impact on both environmental quality and human health. Regarding occupational health, exposure to VOCs poses substantial risks to workers in relevant industries [15,16]. VOCs can infiltrate the human body through inhalation and dermal absorption, potentially leading to various health issues and causing long-term or irreversible harm [17]. Studies have revealed elevated hazard quotients for benzene and chloroform in electro-mechanical repair and automotive painting centers in Argentina [18], heightened exposure levels to benzene, toluene, and paraxylene among workers in a furniture factory in Colombia compared to control groups [19], and a significantly elevated non-carcinogenic risk of acrolein for employees in a furniture shop in Xi’an, China, surpassing acceptable levels by 283 times [20].

Guangdong Province, as an industrial and economic stronghold of China, has organic solvent poisoning cases accounting for more than 70% of its total cases of occupational poisoning [21], and this organic solvent poisoning demonstrates enterprise aggregation, commonly found in the manufacturing industry, especially in light industry and the electronic industry [22]. Bao’an District is an industrially important area in Shenzhen, Guangdong Province, with numerous frontline enterprises. It has been reported that the cases of occupational chemical poisoning accounted for a quarter of the incidence of emerging occupational diseases in this district during the period 2006–2021, most of which were due to benzene, n-hexane and trichloroethylene poisoning [23], with ensuing massive economic losses [24]. Additionally, in the United States, 12.1% of the workforce is occupationally exposed to organic solvents [25], with 87% of methylene chloride-related fatalities identified in occupational settings [26]. Therefore, occupational hazards caused by organic solvents need to be given more attention.

The risk of occupational exposure to organic solvents may vary due to operational site factors, such as differences in processing materials [27], ventilation conditions [28], exposure duration [29], and indoor and outdoor microenvironments [30]. Current studies have focused on occupational exposure to VOCs in individual industries, such as the electronics industry [31], wooden furniture manufacturing industry [32], painting industry [27], printing industry [33], and automobile repair industry [34], while there are comparatively fewer studies that have made cross-sectional comparisons between various categories of industries. In addition, considering the occupational disease-accelerating properties of organic solvents and the introduction of new materials and technologies, concern for the dynamics of organic solvent use and occupational exposure over specific time scales can generate more targeted recommendations for occupational disease prevention and control strategies. As mentioned above, it is imperative to carry out long-term continuous monitoring of organic solvent components in the workplaces of different industries, which can provide a more reliable basis for the selection of organic solvents and the supervision of occupational hazards in enterprises.

Therefore, this study aims to analyze the use of organic solvents and VOCs in different industries in Bao’an District, Shenzhen, China, from 2018 to 2023, to elucidate the temporal variation of volatile components in organic solvents and their industry-specific differences. At the same time, the HQ values of high-risk VOCs are calculated through air monitoring samples to estimate health risks to the human body and identify high-risk occupational hazards.

## 2. Materials and Methods

### 2.1. Samples Collection

The organic solvent samples were collected from routine testing and evaluation at the Yanluo Branch Center for Public Health Services in Bao’an District from 2018 to 2023, including 1335 samples used by 414 different industry enterprises. Among these enterprises, there are 142 enterprises from the electronics industry, 141 from the chemical industry, 92 from light industry, and 39 from the machinery industry.

During sampling, the staff randomly selected organic solvents from the industries’ inventory and collected samples with 50 mL wide mouthed glass sample bottles. After collection, the bottles were sealed and transported back to the Yanluo Branch Center for Public Health Services and stored in the dark at room temperature for further analysis.

### 2.2. Analysis of VOCs

According to the Technical Guidelines of Guangdong Occupational Health Technology Quality Control Center (GDOHTOC 001–2020) [35], VOCs were qualitatively and semi-quantitatively analyzed using the Agilent 7890A-5975C gas chromatography–mass spectrometry (GC–MS) system and a DB-5MS column (60 m × 0.25 mm × 1.00 μm) with the headspace method. Five milliliters (or 5 g) of each sample were placed into a headspace vial and heated at 40 °C for 30 min for equilibration, then 100 µL of the liquid is injected for analysis. Chromatographic conditions: injection port temperature is at 260 °C, initial temperature is at 45 °C holding for 2 min, then increased at a rate of 10 °C/min to 230 °C, holding for 2 min. In the subsequent analysis, we will use peak area percentage (PAP) for comparison. PAP is an analytical method used to describe the relationship between peak size and concentration in chemical analysis. In chemical analysis, peak area refers to the area under the curve of a specific peak, while concentration refers to the concentration of solutes in a solution. By calculating the peak area percentage, the concentration of solutes in the solution can be determined.

### 2.3. Air Detection

Based on recent occupational disease occurrences related to organic solvents, this research regards benzene, 1,2-dichloroethane, n-hexane, trichloroethylene, toluene, ethylbenzene, xylene, and trichloromethane as high-risk occupational hazards [36,37,38] and calculates the health risks of these VOCs. The air samples from different industry enterprises were collected in 2023 for daily monitoring, with a total of 1554 samples, including 522 from the electronics industry, 504 from the chemical industry, 372 from light industry, and 156 from the machinery industry. Each sample was collected by an activated carbon tube with a flow rate of 50 mL/min for 2–8 h at the sampling point. The air samples were analyzed by 7890A gas chromatograph (Agilent, Santa Clara, CA, USA) equipped with flame ionization detector. The activated carbon in the front and rear sections was poured into two solvent desorption bottles and 1.0 mL of carbon disulfide added to each. Then, the bottles were sealed and desorbed for 30 min and shaken occasionally. This solution was later used for detection. The injection volume is 1 μL. The specific reference methods are shown in Table S1.

### 2.4. Human Health Risk Assessment

Health risk assessment can quantitatively describe the relationship between human exposure to VOCs and adverse health reactions. The level of VOC inhaled by the human body is calculated based on the environmental concentration of each type of VOC. Referring to previous literature [39], the exposure concentration EC_i_ (mg/m^3^) in this study was calculated as follows with Formula (1):

(1) $${\mathrm{EC}}_{i}=C_{i}\frac{\mathrm{ET}\;\times \;\mathrm{EF}\;\times \;\mathrm{ED}}{\mathrm{AT}}\;\times \;90\%$$

C_i_ is the environmental concentration of VOCs_i_ (mg/m^3^); ET is the exposure time (8 h/day); EF is the exposure frequency (240 day/year); ED is the lifetime exposure time, which is 30 years; and AT is the average exposure time (70 years × 365 days/year × 24 h/day). The absorption coefficient in the formula is 90%.

The calculation formula for non-carcinogenic risk assessment is as follows using Formula (2):

(2) $${\mathrm{HQ}}_{i}=\frac{{\mathrm{EC}}_{i}}{{\mathrm{REC}}_{i}}$$

The HQ_i_ represents the hazard quotient associated with compound i, while RFC_i_ is the reference concentration (mg/m^3^) of compound i. Based on the non-carcinogenic risk assessment methodology employed by the U.S. Environmental Protection Agency (EPA), it is determined that a non-carcinogenic health risk is present when HQ > 1. When HQ < 1, it indicates that the level of risk is within an acceptable range. Since the EPA did not calculate the RFC of 1,2-dichloroethane and trichloromethane, the calculation of HQ values does not involve these two VOCs.

### 2.5. Statistical Analysis

Statistical analysis is conducted using IBM SPSS Statistics 26. The chi-square test is employed to examine differences in the composition ratio of organic solvent samples across different industries and the detection rates of VOCs in organic solvents. For the volume fraction of VOCs in organic solvents, if homogeneity of variance is satisfied, One-Way ANOVA is applied; if not satisfied, Welch’s ANOVA is used. The significance level for testing is set at α = 0.05.

## 3. Results and Discussion

### 3.1. Overview of VOC Testing Industry and Sample Analysis

#### 3.1.1. Numbers of Organic Solvent Samples in Four Industries

According to the industrial classification for national economic activities (GB/T 4754-2017) [40] and the table of business scope of occupational health technical service organizations [2022 Edition], a total of 414 enterprises in the Yanluo and Songgang Street areas of Bao’an District conducted organic solvent testing from 2018 to 2023. These enterprises represent 16 specific industries, which are further categorized into four major categories: electronics industry, chemical metallurgy and building materials industry (referred to as “chemical industry”), light and textile industry (referred to as “light industry”), and machinery industry. Detailed information regarding these industries is provided in Table S2.

**Figure 1:**
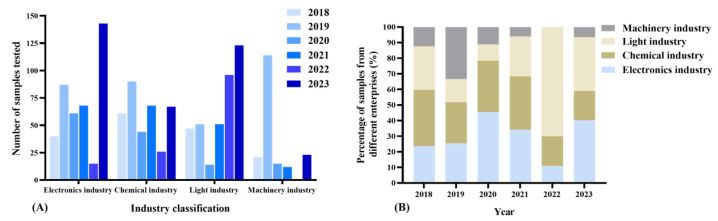
The number (**A**) and proportion (**B**) of organic solvent samples across different industries from 2018 to 2023.

Between 2018 and 2023, a total of 1335 organic solvent samples were analyzed within the jurisdiction. Significantly, the number of organic solvent samples detected notably increased in 2023, particularly within the electronics and light industry sectors (Figure 1A). Conversely, there was minimal change observed in the chemical and mechanical industries (Table S3). This discrepancy may stem from the traditional nature of these industries, which often employ specific types of organic solvents with extended service life and stability, thereby requiring less frequent replacement or testing [41]. Conversely, since the onset of the epidemic, the electronics and light industry sectors have experienced rapid expansion [42,43]. These sectors extensively utilize organic solvents in their production processes, resulting in heightened demand for solvent testing [44]. Regarding the composition ratio of samples from various industries, the majority of organic solvent testing samples in the jurisdiction originated from the electronics industry (10.95–45.52%) and the chemical industry (18.82–36.09%) (Figure 1B, Table S3).

#### 3.1.2. Composition of Organic Solvent Samples from Four Industries

**Figure 2:**
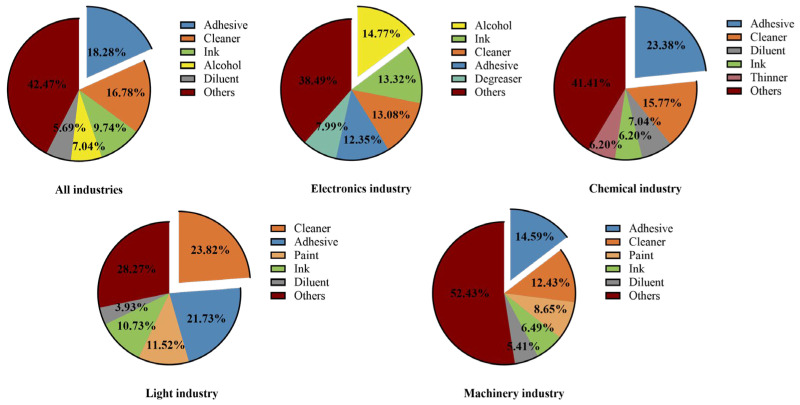
Composition of organic solvents in different industries’ samples.

Among all the samples analyzed, the top five compositions were adhesive (18.28%), cleaner (16.78%), ink (9.74%), alcohol (7.04%), and diluent (5.69%) (Figure 2). The common VOC composition of these five organic solvents is shown in Table S4. Adhesive and cleaner find extensive applications across various industries. Adhesives serve as crucial joining materials in the assembly and manufacturing of diverse products [45], thus accounting for their significant presence in the samples. Cleaners play a vital role in equipment and product cleaning processes [46], underscoring their indispensable nature in manufacturing operations, which contributes to their high representation in the sample composition.

In the electronics industry, alcohol comprises the largest percentage in the sample composition (14.77%), followed by ink (13.32%) (Figure 2). Alcohol, characterized by high volatility and low surface tension, facilitates rapid evaporation [47], effectively removing surface oils and impurities to enhance the cleanliness and reliability of components. Hence, it is utilized extensively for cleaning electronic equipment. On the other hand, ink is employed in the fabrication of printed circuit boards (PCBs) to delineate circuit lines and conductive patterns [48]. Additionally, it finds application in the production of flexible circuits, touchscreens, and other electronic components owing to its conductivity, rendering it indispensable in the electronics industry [49].

### 3.2. Analysis of VOCs in Organic Solvents

#### 3.2.1. Categories of VOCs in Organic Solvents

**Figure 3:**
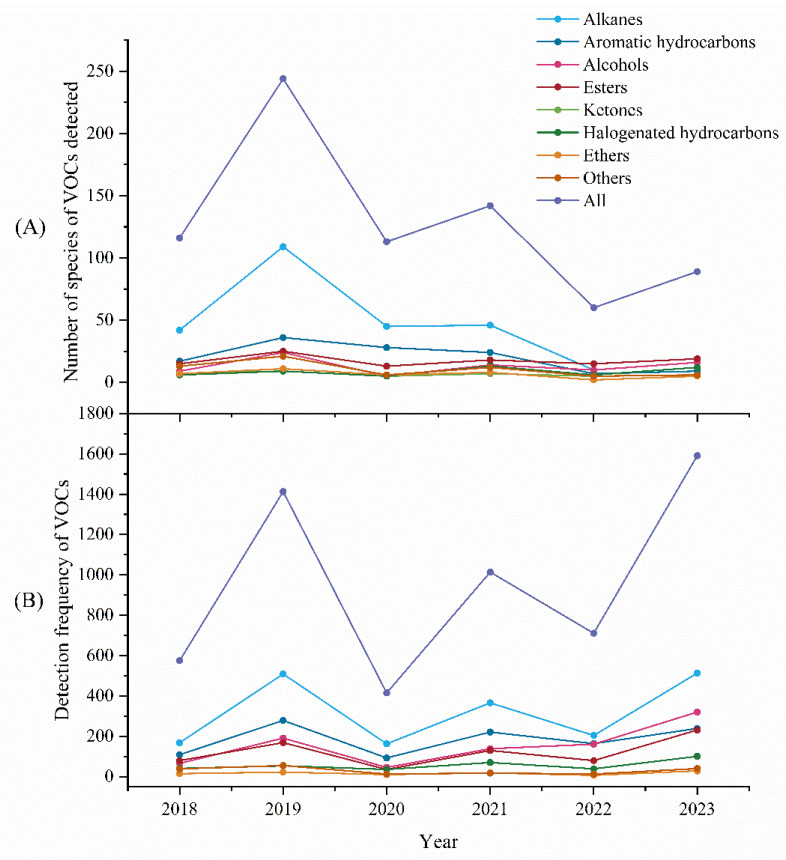
The number (**A**) and detection frequency (**B**) of VOCs in various organic solvents from 2018–2023.

During 2018–2023, a total of 400 VOCs were detected in 1335 organic solvent samples, including 142 alkanes, 53 aromatic hydrocarbons, 48 esters, 38 alcohols, 11 ketones, 20 halogenated hydrocarbons, 20 ethers, and 68 other species, of which 66 were listed in the Occupational exposure limits for hazardous agents in the workplace (GBZ 2.1-2019) [50]. As shown in Figure 3 and Table S5, VOCs were detected 5719 times, and the composition ratios of different components was in the following order: alkanes (33.6%) > aromatic hydrocarbons (19.3%) > alcohols (16.1%) > esters (12.7%) > ketones (7.3%) > halogenated hydrocarbons (6.0%) > others (3.2%) > ethers (1.8%). Overall, the number of alkanes detected (F = 7.346, *p* < 0.01) and their detection frequency (F = 11.925, *p* < 0.01) were significantly higher than those of other VOCs, followed by aromatic hydrocarbons, which was similar to the organic solvent determinations in the other districts of Shenzhen [51,52,53].

As shown in Figure 3, the number of detected VOCs and the detection frequency reached a peak in 2019 and a trough in 2020, which is attributed to the outbreak of the COVID-19 pandemic in 2020, causing the shutdown of many industrial operations, which reduced the demand for organic solvents. Notably, after 2020, there is an overall downward trend in the number of detected VOC categories, but an upward trend in the detection frequency. The calculated results show that the ratios of the detection frequency of VOCs to the total number of detected categories for each year are as follows: 17.9 in 2023 > 11.89 in 2022 > 7.14 in 2021 > 5.79 in 2019 > 4.96 in 2018 > 3.67 in 2020, suggesting that the ratio is increasing year by year after 2020. Evidently, there is a trend towards homogenization with the gradual reduction and centralization of the types of organic solvents applied in industrial production. This reduces the potential for serious risks associated with mixed exposures, while the introduction of new substitutes may raise new health concerns and require further research into the hazards of organic solvents and their occupational exposure risks.

#### 3.2.2. Detection of VOCs in Organic Solvents

The ten most frequently detected VOCs in the 1335 organic solvent samples during 2018–2023 are displayed in Table 1, and they have all been listed in the Occupational exposure limits for hazardous agents in the workplace (GBZ 2.1-2019) [50]. The five substances with the highest detection rates were in the following order: methanol (31.1%) > toluene (22.5%) > n-hexane (22.0%) > xylene (16.1%) > ethylbenzene (15.3%). Among them, toluene (22.1%) exhibited the highest average peak area percentage, followed by methanol (16.9%), n-hexane (4.96%), xylene (0.25%), and ethylbenzene (0.33%). They are all high-risk occupational hazards of organic solvents, especially benzene derivatives with carcinogenic risk, and their detection rate is the main concern of the many studies on organic solvent analysis in Shenzhen [52,54,55,56], requiring key attention in occupational health monitoring.

In the present study, methanol was the most frequently detected and was also the major VOC of various organic samples, including inks, alcohols, diluents, degreaser, thinner, and screen wash. It has been reported that methanol poisoning has a high mortality rate among survivors and can lead to long-term visual sequelae and severe brain damage [57,58,59]. Since methanol can be absorbed by humans through various routes, such as dermal contact, ingestion and inhalation, corresponding protective measures must be taken, such as ventilation and wearing appropriate protective equipment, to avoid prolonged exposure to methanol vapors. It is noteworthy that a relatively low detection rate (6.67%) and mean peak area percentage (0.36%) of benzene were observed in this study. The almost complete cessation of benzene’s industrial use as a solvent has allowed its congeners, such as toluene and xylene, to be introduced into industrial production as low-toxicity alternatives, but benzene remains as a raw ingredient in other materials, such as styrene [60]. However, chronic exposure to low ambient concentrations of benzene still significantly increases the risk of death in the population [61]. Furthermore, beyond the well-known carcinogenicity and hematologic toxicity of benzene, toluene, ethylbenzene, and xylene (BTEX) [27], epidemiological studies have demonstrated associations between BTEX exposure and cardiovascular disease [62], decreased lung function [63], liver damage [64], and neurological symptoms [65]. n-Hexane is frequently applied as an auxiliary production material in small and medium-sized enterprises, especially in the Pearl River Delta region where the electronics, hardware and printing industries are concentrated, and it has been associated with peripheral neuropathy [29]. More remarkably, workers are subject to combined exposures to various hazards that may exacerbate occupational injuries when laboring in workshops. It has been reported that exposure to mixtures of organic solvents is related to the prevalence of hypertension [66], and that the combined effects of organic solvents and noise have a synergistic effect on hearing damage [67,68,69].

### 3.3. Detection and Analysis of High-Risk Occupational Disease Hazards

#### 3.3.1. Detection of High-Risk Occupational Hazards in Organic Solvents from 2018 to 2023

Table 2 shows the detection rates and average peak area percentages of high-risk VOCs of organic solvents from 2018 to 2023. Significant differences were found in the detection rates of 8 VOCs (χ^2^ = 741.120, *p* < 0.001). Over the past six years, toluene (22.47%), n-hexane (22.02%), ethylbenzene (15.28%), and xylene (16.10%) had higher detection rates, while the detection rates of trichloromethane (0.60%), 1,2-dichloroethane (1.05%), trichloroethylene (5.54%), and benzene (6.67%) were lower. Additionally, the average peak area percentages of toluene (14.48%) and xylene (12.93%) were significantly higher than other VOCs (F = 10.119, *p* < 0.001) (Table 2), as substitutes for benzene, toluene and xylene have been widely used in various industries in recent years [70,71]. Although the toxicity of toluene and xylene is lower than that of benzene, studies have also shown that long-term exposure to toluene and xylene is associated with cardiovascular diseases and can lead to premature births [62,72]. Therefore, the use of toluene and xylene needs to be cautious, and efforts should continue to find alternatives with lower toxicity.

Between 2018 and 2023, the annual detection rates of benzene, 1,2-dichloroethane, toluene, ethylbenzene, xylene, and trichloromethane fluctuated within a certain range. Benzene (12.41%), toluene (37.23%), ethylbenzene (28.47%), and xylene (29.20%) all saw their highest detection rates in 2022 (Table 2). It is worth noting that the detection rate of n-hexane increased from 9.47% in 2018 to 32.02% in 2023, showing an upward trend over the past six years. n-Hexane is an industrial organic solvent, and its toxicity is mainly neurotoxic. Prolonged exposure to n-hexane can induce severe peripheral neuropathy, and it is also associated with central-peripheral axonopathy [73,74]. Meanwhile, the detection rate of trichloroethylene decreased from 9.47% in 2018 to 2.53% in 2023, showing a downward trend over the past six years. Trichloroethylene is also a commonly used industrial solvent, its toxicity may lead to congenital defects and neurodegenerative diseases, and it is considered a potential risk factor for the development of Parkinson’s disease (PD) [75,76]. The gradual decrease in the detection rate of trichloroethylene in recent years indicates that other solvents have gradually been used to replace it in industry. Although the usage rate has declined, its toxicity still cannot be ignored.

#### 3.3.2. Detection of High-Risk Occupational Hazards in Organic Solvents Used in Different Industries

Table 3 shows the detection rates and average peak area percentages of high-risk VOCs in different industries. Among the 414 enterprises, samples from the electronics industry, chemical industry and light industry were able to detect all eight high-risk VOCs. In the machinery industry, all VOCs except trichloromethane were detected. The detection rates of n-hexane (13.11~24.94%), toluene (12.02~32.2%), ethylbenzene (7.26~21.99%), and xylene (8.23~22.25%) are higher than for other VOCs. 

The average peak area percentage of VOCs represents the volumetric fraction of VOCs. According to Table 3, we can see that the volumetric fraction of toluene (14.96~24.69%) is relatively high in each industry, while the volumetric fraction of benzene (0.1~1.32%) is lower than other VOCs. The volumetric fractions of 1,2-dichloroethane (19.5%), trichloroethylene (55.61%), and xylene (43.76%) in the mechanical industry are higher than in other industries. In addition, the volumetric fraction of trichloroethylene (54.89%) in the chemical industry is also higher than in other industries. The volumetric fraction of trichloromethane (35.71%) in the light industry is higher than in other industries. This study detected the content of VOCs in organic solvents used in industries, while most other literature detected the content of VOCs in factory air. Although the samples are different, the detection levels also have certain reference value. Xie et al. detected the types and levels of VOCs in the air from the petrochemical industry in China. The results indicate that benzene and n-hexane are characteristic VOCs of the petrochemical industry (mass content: 14.54% and 4.24%) [77]. Another piece of research from Jinan, China shows that, besides benzene (average mixing ratio: 1.35 ± 1.32 ppbv) and n-hexane (0.92 ± 0.49 ppbv), there are also higher levels of toluene (1.46 ± 1.34 ppbv) and xylene (1.51 ± 1.57 ppbv) in the air from the petrochemical industry [78]. Based on the results of this study and other related research, we suggest that, in order to control the hazards of VOCs to human health, it is necessary for various industries to reduce the usage of benzene, toluene, xylene, and ethylbenzene, and continue to search for low-toxicity alternatives for these compounds.

### 3.4. The Health Risk of High-Risk Occupational Hazards

#### 3.4.1. Detection of High-Risk Occupational Hazards in Air Samples

Table 4 shows the detection rate and mean concentration of high-risk VOCs in air samples from different enterprises in 2023. Except for 1,2-dichloroethane in the chemical industry and 1,2-dichloroethane and trichloromethane in the machinery industry, all VOCs have been detected in all industries. According to Table 4, the DRs of benzene, 1,2-dichloroethane, and trichloromethane are relatively low, while the DRs of n-hexane, toluene, and xylene are all around 50%. The detection rate of toluene in air samples from the light industry is as high as 71.15%, and the detection rate of toluene in the organic solvent samples from the light industry is also the highest (32.2%), indicating that the use of toluene in light industry is relatively high. It is necessary to provide targeted protection for workers in light industry to reduce the harm of toluene to them.

In air samples, the mean concentration of trichloroethylene is significantly higher than that of other VOCs, but its detection rate in organic solvent samples is not the highest. This indicates that the volatility of trichloroethylene is relatively high in all industries, and it may also be due to improper ventilation and exhaust protection measures in workshops using trichloroethylene. In addition to trichloroethylene, the mean concentrations of n-hexane, toluene, and xylene in some industries are also relatively high. A study of the petrochemical industry in Map Ta Phut, Thailand, found that the highest annual ambient concentration of xylene (57.9 µg/m^3^) in the air is approximately 6 to 20 times higher than benzene’s (9 µg/m^3^) and toluene’s (2.8 µg/m^3^) [79]. Compared to research in Thailand, the exposure to VOCs in Shenzhen is significantly more severe. The research on the pharmaceutical industry in the Yangtze River Delta in China [80], the manufacturing industries in Dalian, Shenyang, and Kaifeng in China [81], and the paint production plants in Iran [82] all show that benzene, xylene, and ethylbenzene are the most prominent VOCs. These types of VOCs have a certain toxicity to the human body, especially neurotoxicity [75,83,84,85]. Therefore, industries should enhance health monitoring of workers and, if workers have early symptoms, immediate action should be taken to avoid further exacerbation of poisoning.

#### 3.4.2. The Health Risk of High-Risk Occupational Hazards in Different Industries

Table 5 shows the health risks of high-risk volatile organic compounds in different industries in 2023. For all industries, exposure to benzene, n-hexane, toluene, and ethylbenzene poses no risk to human health (HQ < 1). Xylene poses a certain risk to the health of workers in the Electronics and Light industries (3 < HQ < 10), while its harm in the other two enterprises is several times greater (HQ > 10). The health hazards of trichloroethylene for all industrial workers are significant (HQ > 10), indicating that Shenzhen industries need to impose stricter restrictions on the use of trichloroethylene.

Previous studies have shown that xylene poses almost no health risk to humans in petrochemical plants in Iran [86] and industrial zones in Chongqing [87] and Hefei [88], China (HQ < 1). However, in other industries, the results of benzene, toluene, ethylbenzene, and xylenes (BTEX) are completely different. In Iranian refineries, BTEX in the air poses a health risk to humans (1 < HQ < 5) [89,90]. In printing factories in Beijing, the health risks of benzene and xylene cannot also be ignored (1 < HQ < 5) [91]. However, compared with other studies, the health risks of trichloroethylene and xylene in this study are still higher, indicating that the usage of trichloroethylene and xylene in Shenzhen industry is relatively high, and the prevention and control measures are not timely. At present, the permissible concentration-time weighted average (PC-TWA) of xylene is 50 mg/m^3^ in China, while the PC–TWA of trichloroethylene is 30 mg/m^3^. We can see that industries in Shenzhen all follow the national limit for the use of xylene, but the usage of trichloroethylene is over the limit. We suggest that factories should strengthen ventilation measures and reduce the use of these two types of VOCs. For trichloroethylene, factories should reduce its usage below the national limit, while the limit value of xylene should be appropriately lowered, as the current limit value cannot effectively reduce the health hazards of xylene for workers. Factories should regularly monitor the concentration of these in the air and conduct timely physical examinations of workers to minimize the adverse health effects of trichloroethylene and xylene.

## 4. Conclusions

This study detected 1335 organic solvent samples used by 414 different industry enterprises in Bao’an District from 2018 to 2023. The result shows that, during the outbreak of the pandemic, most industries reduced their use of organic solvents. However, after the end of the pandemic, rapid expansion due to economic demands led to an increased demand for organic solvents. At the same time, with a trend towards the homogenization of organic solvent types in industrial production, this has reduced the associated risks of mixed exposure. Among all detected VOCs, methanol has the highest detection rate. However, among the eight high-risk VOCs, toluene, ethylbenzene, xylene, and n-hexane have relatively high detection rates. Trichloroethylene and xylene pose high health risks to workers (HQ > 1), and industries should strengthen monitoring of these two VOCs. 

This study also has certain limitations, the most important one is that no biological samples were collected from workers for detection, making it impossible to directly determine the exposure effects of VOCs on workers. We only use HQ values to reflect the potential hazards of VOCs to workers. The hazards of VOCs and their occupational exposure risks should continue to be given attention to, with ongoing research efforts aimed at gaining a deeper understanding.

## Figures and Tables

**Table 1:** Top ten volatile organic compounds detected in 1335 organic solvent samples.

NO.	Components	DF	DR (%)	PAP (%)	
				Mean	Range
1	Methanol	415	31.1	16.9	0.10–100
2	Toluene	300	22.5	22.1	0.10–100
3	n-Hexane	294	22.0	4.96	0.01–41.4
4	Xylene	215	16.1	0.25	0.10–99.1
5	Ethylbenzene	204	15.3	0.33	0.10–56.1
6	Ethyl acetate	196	14.7	12.9	0.10–100
7	Dichloromethane	182	13.6	25.8	0.10–100
8	Methyl acetate	174	13.0	9.57	0.10–92.5
9	Dimethoxymethane	169	12.7	13.3	0.10–82.9
10	Acetone	168	12.6	11.1	0.10–100

DF: detection frequency, the times that VOCs were detected; DR: detection rates, the proportion of VOCs detected in all samples; PAP: peak area percentage, the proportion of the peak area to the total peak area.

**Table 2:** High-risk volatile organic compounds from 2018 to 2023.

Years	Benzene		1,2-Dichloroethane		n-Hexane		Trichloroethylene	
	DR (%)	APAP (%)	DR (%)	APAP (%)	DR (%)	APAP (%)	DR (%)	APAP (%)
2018 (*n* = 169)	5.92	0.09	0.59	1.39	9.47	3.92	9.47	13.82
2019 (*n* = 340)	4.41	0.63	1.18	1.59	13.53	6.05	5	9.78
2020 (*n* = 134)	2.99	0.02	0.75	0.34	15.67	2.08	8.21	4.99
2021 (*n* = 199)	6.53	0.04	1.51	0.58	27.64	1.77	8.04	5.13
2022 (*n* = 137)	12.41	0.03	1.46	0.01	30.66	1.53	3.65	1.24
2023 (*n* = 356)	8.43	0.11	0.84	0.01	32.02	2.42	2.53	1.76
all samples (*n* = 1335)	6.67 ^a^	0.16 ^1^	1.05 ^b^	0.70 ^1^	22.02 ^c^	2.80 ^1^	5.54 ^a^	7.38 ^1^
**Years**	**Toluene**		**Ethylbenzene**		**Xylene**		**Trichloromethane**	
	**DR (%)**	**APAP (%)**	**DR (%)**	**APAP (%)**	**DR (%)**	**APAP (%)**	**DR (%)**	**APAP (%)**
2018 (*n* = 169)	17.75	15.46	10.65	1.91	15.98	21.5	0	0
2019 (*n* = 340)	21.47	25.45	16.18	4.91	12.94	22.01	0	0
2020 (*n* = 134)	18.66	13.58	7.46	0.62	7.46	2.89	0.75	1.53
2021 (*n* = 199)	27.14	9.63	21.11	2.75	21.11	10.52	1.01	1.93
2022 (*n* = 137)	37.23	12.09	28.47	2.51	29.2	8.29	0.73	4.17
2023 (*n* = 356)	18.82	8.16	11.24	3.02	14.61	8.26	1.12	0.43
all samples (*n* = 1335)	22.47 ^c^	14.48 ^2^	15.28 ^d^	3.16 ^1^	16.10 ^d^	12.93 ^2^	0.60 ^b^	1.41 ^1^

DR: detection rates; APAP: average peak area percentage; ^a, b, c, d^: Different superscript letters indicate *p* < 0.001 (Chi-square test); ^1, 2^: Different superscript numbers indicate *p* < 0.001 (One-Way ANOVA).

**Table 3:** High-risk volatile organic compounds in different industries.

Industry Classification	Benzene		1,2-Dichloroethane		n-Hexane		Trichloroethylene	
	DR (%)	APAP (%)	DR (%)	APAP (%)	DR (%)	APAP (%)	DR (%)	APAP (%)
Electronics industry (*n* = 413)	4.84 ^a^	0.19	0.48 ^b^	0.09	24.94 ^c^	4.38	8.96 ^a,d^	27.47
Chemical industry (*n* = 355)	6.76 ^a^	0.22	0.85 ^b^	7.48	24.23 ^c,d^	6.44	5.92 ^a^	54.89
Light industry (*n* = 382)	8.90 ^a^	0.1	2.09 ^b^	2.17	21.47 ^c^	2.86	1.31 ^b^	0.48
Machinery industry (*n* = 185)	6.01 ^a,b^	1.32	0.55 ^a^	19.5	13.11 ^b^	8.2	2.73 ^a^	55.61
**Industry classification**	**Toluene**		**Ethylbenzene**		**Xylene**		**Trichloromethane**	
	**DR (%)**	**APAP (%)**	**DR (%)**	**APAP (%)**	**DR (%)**	**APAP (%)**	**DR (%)**	**APAP (%)**
Electronics industry (*n* = 413)	12.35 ^d^	14.96	7.26 ^a,d^	2.55	8.23 ^a,d^	10.97	0.48 ^b^	3.48
Chemical industry (*n* = 355)	28.73 ^d^	24.69	18.59 ^c^	5.91	21.69 ^c,d^	21.9	0.85 ^b^	8.98
Light industry (*n* = 382)	32.2 ^d^	24.15	21.99 ^c^	3.66	22.25 ^c,d^	16.27	0.79 ^b^	35.71
Machinery industry (*n* = 185)	12.02 ^b^	21.12	12.57 ^b^	11.71	10.93 ^b^	43.76	0.00 ^a^	0

DR: detection rates; APAP: average peak area percentage; ^a, b, c, d^: Different superscript letters indicate *p* < 0.001 (Chi-square test).

**Table 4:** High-risk VOCs in air samples from different industries in 2023.

Industry Classification	Benzene		1,2-Dichloroethane		n-Hexane		Trichloroethylene	
	DR (%)	Mean (mg/m^3^)	DR (%)	Mean (mg/m^3^)	DR (%)	Mean (mg/m^3^)	DR (%)	Mean (mg/m^3^)
Electronics industry (*n* = 522)	10.59	0.18	7.84	0.13	40.23	2.82	7.59	105.89
Chemical industry (*n* = 504)	11.54	0.12	0	-	43.04	4.21	24.20	145.02
Light industry (*n* = 372)	16.67	0.03	3.85	0.03	56.67	2.50	5.13	54.56
Machinery industry (*n* = 156)	16.67	0.03	0	-	50.00	10.73	16.67	175.68
**Industry classification**	**Toluene**		**Ethylbenzene**		**Xylene**		**Trichloromethane**	
	**DR (%)**	**Mean (mg/m^3^)**	**DR (%)**	**Mean (mg/m^3^)**	**DR (%)**	**Mean (mg/m^3^)**	**DR (%)**	**Mean (mg/m^3^)**
Electronics industry (*n* = 522)	34.85	4.70	25.42	2.00	42.30	6.64	13.04	6.54
Chemical industry (*n* = 504)	53.62	24.52	43.55	3.27	60.00	13.66	5.88	0.20
Light industry (*n* = 372)	71.15	18.60	45.83	2.04	50.00	3.87	6.25	0.04
Machinery industry (*n* = 156)	50.00	10.04	40.00	3.36	50.00	30.85	0	-

DR: detection rates.

**Table 5:** The HQ of high-risk VOCs in different industries in 2023.

Industry Classification	Benzene	n-Hexane	Trichloroethylene	Toluene	Ethylbenzene	Xylene
Electronics industry	0.51	0.09	>10 ^a^	0.08	0.06	5.61
Chemical industry	0.34	0.14	>10 ^a^	0.41	0.10	>10 ^a^
Light industry	0.08	0.08	>10 ^a^	0.31	0.06	3.27
Machinery industry	0.08	0.35	>10 ^a^	0.17	0.10	>10 ^a^

^a^: When the HQ value is much greater than 10, use >10 instead of the true value.

## Data Availability

Data are available from the corresponding author by request.

## Supplementary Materials

The following supporting information can be downloaded at https://www.mdpi.com/article/10.3390/toxics12090634/s1, Table S1. Detection-related information for high-risk VOCs; Table S2: Industry classification and sectors; Table S3: Enterprise and sample situation of organic solvent detection in different industries from 2018 to 2023; Table S4: The top ten VOCs with high detection rates in common organic solvent samples; Table S5: Detection of volatile components in different categories of organic solvents from 2018–2023.
