# Supplementary material for: Temporal Variation and Industry-Specific Differences of the Use of Volatile Organic Compounds from 2018 to 2023 and Their Health Risks in a Typical Industrially Concentrated Area in South China
Source: Toxics. 2024 Aug 29;12(9):634. doi: 10.3390/toxics12090634 (PMC11436172; doi:10.3390/toxics12090634)
Supplement: Supplementary file 1 [file toxics-12-00634-s001.zip › toxics-3107424-supplementary.pdf]

Supplementary materials

Temporal variation and industry-specific differences of the use of volatile organic compounds from 2018 to 2023 and their health risks in a typical industrially concentrated area in south China

Yijia Guo [1], Lihua Zhu [1], Liyin Zhang [1], Xinxin Tang [2], Xinjie Li [2], Yiming Ge [2], Feng Li [1], Jilong Yang [1,3], Shaoyou Lu [2], Jinru Chen [1*] and Xiaotao Zhou[1*]

[1] Public Health Service Center, Bao'an District, Shenzhen, 518126, China; beckyguo1995@126.com (Yijia Guo); zhuivy810@126.com (Lihua Zhu); 13798509754@126.com (Liyin Zhang); lf13424232209@163.com (F.L.); yangjlong6@mail2.sysu.edu.cn (J.Y.)

[2] School of Public Health (Shenzhen), Shenzhen Campus of Sun Yat-sen University, Shenzhen, 518107, China; tangxx26@mail2.sysu.edu.cn (X.T.); lixj328@mail2.sysu.edu.cn (X.L.); geym5@mail2.sysu.edu.cn (Yiming Ge); lushy23@mail.sysu.edu.cn (S.L.)

[3] School of Public Health, Sun Yat-Sen University, Guangzhou, 510080, China

* Correspondence: sgzjk209@126.com (J.C.); cybersoul@126.com (X.Z.); Tel.: +86-139-2745-0193 (J.C.); +86-186-1718-7906 (X.Z.)

Number of pages (including this page): 6

Number of tables: 5

Table S1. Detection-related information for high-risk VOCs.

Components	LOD (mg/m^3)	LOQ (mg/m^3)	Detecting standard
Benzene	0.02	0.11	GBZ/T 300.66-2017
1,2-Dichloroethane	0.04	0.20	GBZ/T 160.45-2007
n-Hexane	0.04	0.20	GBZ/T 300.60-2017
Trichloroethylene	0.23	1.33	GBZ/T 300.78-2017
Toluene	0.03	0.13	GBZ/T 300.66-2017
Ethylbenzene	0.25	0.35	GBZ/T 300.66-2017
Xylene	0.25	0.35	GBZ/T 300.66-2017
Trichloromethane	0.08	1.13	GBZ/T 300.73-2017

LOD: Limit of detection;

LOQ: Limit of quantitation.

Table S2. Industry classification and sectors.

Industry classification	Industry sectors
Electronics industry	Computer, communication, and other electronic equipment manufacturing industry
Chemical, metallurgy, and building materials industry (Chemical industry)	Rubber and plastic products manufacturing industry
	Chemical fiber manufacturing industry
	Chemical raw materials and chemical products manufacturing industry
	Metal products industry
	Non-metallic mineral products industry
Light and textile industry (Light industry)	Paper and paper products manufacturing industry
	Leather, fur, feather and their products and footwear manufacturing industry
	Furniture manufacturing industry
	Cultural, educational, artistic, sporting and recreational products manufacturing industry
	Wood processing and wood, bamboo, rattan, palm and grass products manufacturing industry
	Printing and record media reproduction industry
Machinery industry	Railway, shipbuilding, aerospace, and other transportation equipment manufacturing industry
	Special equipment manufacturing industry
	Electrical machinery and equipment manufacturing industry
	Instrumentation manufacturing industry

Table S3. Enterprise and sample situation of organic solvent detection in different industries from 2018 to 2023.

Province	Tissue	Electronics industry	Chemical industry	Light industry	Machinery industry	Total
2018	enterprise count (composition ratio%)	16 (25.40)	25 (39.68)	13 (20.63)	9 (14.29)	63
	sample size (composition ratio%)	40 (23.67)	61 (36.09)	47 (27.81)	21 (12.43)	169
2019	enterprise count (composition ratio%)	28 (43.75)	25 (39.06)	7 (10.94)	4 (6.25)	64
	sample size (composition ratio%)	86 (25.29)	89 (26.18)	51 (15.00)	114 (33.53)	340
2020	enterprise count (composition ratio%)	23 (37.10)	25 (40.32)	8 (12.90)	6 (9.68)	62
	sample size (composition ratio%)	61 (45.52)	44 (32.84)	14 (10.45)	15 (11.19)	134
2021	enterprise count (composition ratio%)	31 (34.83)	31 (34.83)	19 (21.35	8 (8.99)	89
	sample size (composition ratio%)	68 (34.17)	68 (34.17)	51 (25.63)	12 (6.03)	199
2022	enterprise count (composition ratio%)	5 (12.50)	10 (25.00)	25 (62.50)	0 (0.00)	40
	sample size (composition ratio%)	15 (10.95)	26 (18.98)	96 (70.07)	0 (0.00)	137
2023	enterprise count (composition ratio%)	39 (40.63)	25 (26.04)	20 (20.83)	12 (12.50)	96
	sample size (composition ratio%)	143 (40.17)	67 (18.82)	123 (34.55)	23 (6.46)	356

Table S4. The top ten VOCs with high detection rates in common organic solvent samples.

Adhesive (n=244)		cleaner (n=224)		ink (n=130)		alcohol (n=94)		diluent (n=76)	
VOCs	DR (%)	VOCs	DR (%)	VOCs	DR (%)	VOCs	DR (%)	VOCs	DR (%)
Toluene	44.67	n-Hexane	38.39	Methanol	31.54	Methanol	79.79	Methanol	31.58
n-Hexane	22.13	Dichloro-methane	25.45	Isopropanol	27.69	Ethanol	26.60	Xylene	28.95
Methyl acetate	20.90	n-Heptane	22.77	Toluene	25.38	n-Hexane	20.21	Ethyl acetate	27.63
Acetone	20.08	Methanol	21.43	Xylene	23.08	Dichloromethane	14.89	Toluene	27.63
Methanol	17.21	Cyclohexane	20.09	Ethyl acetate	22.31	Dimethox-ymethane	10.64	Ethylbenzene	26.32
Ethyl acetate	16.80	Methyl pentane	14.29	Acetone	19.23	Toluene	8.51	Dimethox-ymethane	25.00
Ethylbenzene	16.80	n-Pentane	12.05	Ethanol	19.23	Isopropanol	8.51	n-Hexane	18.42
Xylene	16.39	n-Octane	12.05	Ethylbenzene	19.23	Dimethyl ether	7.45	Methyl acetate	17.11
Dichloro-methane	13.93	Trichloroeth-ylene	11.61	n-Hexane	15.38	Ethyl acetate	6.38	Isopropanol	17.11
Cyclohexane	12.30	Ethanol	11.61	Cyclohexa-none	13.85	Methyl pentane	5.32	Butanone	14.47

DR: detection rates, the proportion of VOCs detected in all samples.

Table S5. Detection of volatile components in different categories of organic solvents from 2018-2023.

**Components**	**2018**			**2019**			**2020**			**2021**			**2022**			**2023**			**2018-2023**		
	N	DF	CR (%)	N	DF	CR (%)	N	DF	CR (%)	N	DF	CR (%)	N	DF	CR (%)	N	DF	CR (%)	N	DF	CR (%)
Alkanes	42	168	29.2	109	509	36.0	45	163	39.3	46	366	36.1	10	204	28.7	16	513	32.2	-	1923	33.6
Aromatic hydrocarbons	17	109	19.0	36	279	19.8	28	93	22.4	24	221	21.8	7	164	23.1	9	239	15.0	-	1105	19.3
Alcohols	9	66	11.5	24	191	13.5	5	46	11.1	14	139	13.7	10	161	22.6	16	320	20.1	-	923	16.1
Esters	15	80	13.9	25	169	12.0	13	36	8.7	18	130	12.8	15	80	11.3	19	231	14.5	-	726	12.7
Ketones	7	57	9.9	9	133	9.4	5	17	4.1	7	51	5.0	5	43	6.0	6	118	7.4	-	419	7.3
Halogenated hydrocarbons	6	41	7.1	9	54	3.8	5	36	8.7	13	70	6.9	6	39	5.5	12	101	6.3	-	341	6.0
Ethers	7	15	2.6	11	23	1.6	6	11	2.6	8	19	1.9	2	7	1.0	5	29	1.8	-	104	1.8
Others	13	39	6.8	21	55	3.9	6	13	3.1	12	18	1.8	5	13	1.8	6	40	2.5	-	178	3.2
**All**	116	575	100	244	1413	100	113	415	100	142	1014	100	60	711	100	89	1591	100	400	5719	100

N: the number of volatile components detected;

DF: detection frequency, the times that volatile components were detected;

CR: composition ratio, $CR = \left(\frac{DF\ of\ \ a\ volatile\ component}{DF\ of\ total\ volatile\ components}\right) \times 100\%$.
